# Mass Spectrometry-Based Proteomics for the Analysis of Chromatin Structure and Dynamics

**DOI:** 10.3390/ijms14035402

**Published:** 2013-03-06

**Authors:** Monica Soldi, Alessandro Cuomo, Michael Bremang, Tiziana Bonaldi

**Affiliations:** Department of Experimental Oncology, European Institute of Oncology (IEO), Via Adamello 16, Milan 20139, Italy; E-Mails: monica.soldi@ieo.eu (M.S.); alessandro.cuomo@ieo.eu (A.C.); michael.bremang@ieo.eu (M.B.)

**Keywords:** chromatin, histone post-translational modifications (hPTMs), combinatorial modifications, epigenetics, mass spectrometry, proteomics, SILAC, histone code readers, histone variants

## Abstract

Chromatin is a highly structured nucleoprotein complex made of histone proteins and DNA that controls nearly all DNA-dependent processes. Chromatin plasticity is regulated by different associated proteins, post-translational modifications on histones (hPTMs) and DNA methylation, which act in a concerted manner to enforce a specific “chromatin landscape”, with a regulatory effect on gene expression. Mass Spectrometry (MS) has emerged as a powerful analytical strategy to detect histone PTMs, revealing interplays between neighbouring PTMs and enabling screens for their readers in a comprehensive and quantitative fashion. Here we provide an overview of the recent achievements of state-of-the-art mass spectrometry-based proteomics for the detailed qualitative and quantitative characterization of histone post-translational modifications, histone variants, and global interactomes at specific chromatin regions. This synopsis emphasizes how the advances in high resolution MS, from “Bottom Up” to “Top Down” analysis, together with the uptake of quantitative proteomics methods by chromatin biologists, have made MS a well-established method in the epigenetics field, enabling the acquisition of original information, highly complementary to that offered by more conventional, antibody-based, assays.

## 1. Introduction

Chromatin is a highly ordered nucleoprotein complex that both mediates the DNA compaction into the eukaryotic nucleus and regulates gene expression. At the structural level, the basic unit of chromatin is the nucleosome, consisting of 147bp DNA wound around an octamer core containing one histone H3–H4 tetramer and two histone H2A-H2B dimers [1,2]. Functionally, chromatin is organized into two distinct regions: euchromatin is less condensed and generally permissive for transcription, whereas heterochromatin is highly condensed and transcriptionally silent. Heterochromatin is classified as being either constitutive or facultative. In constitutive heterochromatin, the DNA remains condensed throughout the cell cycle. In facultative heterochromatin however the DNA can lose its condensed form and become transcriptionally active in response to distinct signals [3–5].

Changes in the chromatin structure that do not involve the nucleotide sequence can translate into heritable adjustments of gene expression and thus be stored as an “epigenetic memory” of the cell [6–10]. Epigenetic inheritance can be explained through a step-wise model proposing that “epigenator, initiator and maintainer” factors operate sequentially and synergistically to enforce and maintain specific functional states of the genome [11]. The “epigenator”, a signal emanating from the external environment, is translated by an “initiator” into a specific chromatin/DNA functional state, which is sustained by a number of different “maintainer” factors. These factors include the methylation of cytosine in CpG islands [12,13], covalent post-translational modifications of histones (hPTMs) and, in light of more recent studies, the activities of non-coding RNAs (ncRNA) [14,15].

Among the epigenetic maintainers listed, histone PTMs are largely recognized as key regulators of chromatin structure and function. hPTMs include acetylation, ubiquitination and sumoylation of Lysines; different methylation degrees of Arginines and Lysines; phosphorylation of Serines, Threonines and Tyrosines; ADP-ribosylation of Arginines, Glutamic and Aspartic acids; deimination (or citrullination) of Arginine; Proline isomerization [16–18], and in addition some less-characterized modifications.

The histone code hypothesis proposes that these modifications act either singly or in combination to control distinct downstream pathways or processes on chromatin, ultimately defining the functional status of the underlying DNA. The “letters” of this code are the modifications themselves, which are placed and removed by enzymes known as “writers” and “erasers”, respectively. hPTMs exert their function on chromatin through two distinct mechanisms. In the first, higher order chromatin structure is altered via changes in inter-nucleosomal or histone-DNA interactions, thus controlling the accessibility of DNA-binding proteins such as transcription factors (*cis* mechanisms). Alternatively, hPTMs can generate binding platforms for the recruitment of effector proteins containing specialized domains (*trans* mechanisms): the so-called “readers” of the code. The “readers” translate the information encoded by the modification patterns into specific biological outcomes [19–22]. In addition to hPTM patterns, chromatin is characterized by the local enrichment of a distinct set of histone variants; binding proteins, including various ATP-dependent chromatin remodelling complexes; and differential nucleosome density. Together, these components contribute to the establishment of specific “chromatin landscapes”, defining the functional state of the genome in that territory [23].

Antibodies specifically selected against hPTMs are traditionally used to study the language of histone modification through various assays. These include: immunofluorescence (IF) analyses of modifications at the single cell level, immunoblotting (WB) to profile PTMs in different samples and/or conditions, and chromatin immunoprecipitation (ChIP) that can be coupled to either PCR, DNA microarray (ChIP-on-chip) or deep sequencing (ChIP-Seq) for targeted or large-scale gene expression analysis. The last two methods allow the genome-wide mapping of modifications, with a resolution of a few nucleosomes [24–26]. Although advantageous for their sensitivity, antibody-based assays are hampered by limitations in their specificity and efficiency when used to reveal the combinatorial aspect of the code. In fact, modifications can occur on adjacent or closely spaced residues within the same histone, making an epitope-masking effect more likely. For instance, acetylation of K14 and phosphorylation of S10 co-occur on the H3 *N*-terminal region [27,28]. In this way, the modifications may escape detection by antibodies that are not specifically designed to recognize both modifications on the same epitope. To address this issue, a number of strategies have been developed to assess accurately the specificity of antibodies used in epigenetic research. Peach *et al.* combine immunoprecipitation (IP) of native HPLC-purified H3 with mass spectrometry to detect PTMs co-enriched by a certain antibody on the same polypeptide. Also, Fuchs *et al.* have developed a peptide-array assay, based on a comprehensive library of modified peptides [29,30].

Mass spectrometry (MS) has emerged as a promising complementary analytical strategy to identify known and novel PTMs on proteins, as well as for the relative quantitation and detection of synergies between them [31]. The recent advent of high-resolution mass spectrometry has increased the relevance of MS-based hPTM analysis by enabling the discrimination of near-isobaric modifications, either singly or in combinations, on very long polypeptides and even on intact histones [32–40]. Finally, the use of different labeling strategies, both chemical and metabolic, has enabled the accurate quantitation of modifications, both in a relative and absolute manner [41].

The “epigenomics” and “chromatomics” disciplines share a common goal in studying chromatin structure, composition and features: to gain a comprehensive view, from genome to proteome, of the epigenetic phenomena underlying the establishment and inheritance of specific expression patterns [42,43]. In this review we provide an overview of the contributions made by MS-based proteomics towards achieving this ambitious aim.

## 2. Fundamentals of Mass Spectrometry Technology

Before considering the different MS strategies applied to in-depth investigations of histones and non-histonic chromatin proteins, we offer here a concise synopsis of the basic principles of mass spectrometry, referring to specialized reviews for more detailed descriptions [44,45].

Essentially, all mass spectrometers measure the mass-to-charge ratio (m/z) of freely moving gas-phase ions in electric and/or magnetic fields. One of the most important developments in instrumentation has been the introduction of “soft-ionization” technology, which permits proteins and peptides to be analyzed by MS. Proteins and peptides are polar, nonvolatile species that require an ionization method to transfer them into the gas phase, without extensive degradation. Two techniques paved the way for the modern bench-top MS proteomics: matrix-assisted laser desorption ionization (MALDI) [46,47] and electrospray ionization (ESI) [48]. In a MALDI source, peptides are co-crystallized with a solid-phase matrix onto a metal plate. The matrix typically consists of a small organic molecule such as α-cyano-4-hydroxycinnamic acid or dihydrobenzoic acid (DHB). When laser pulses irradiate the resulting solid mixture, this absorbs the laser energy and transfers it to the acidified peptides. At the same time, the rapid heating causes desorption of both matrix and newly formed [M+H]^+^ protonated peptides into the gas phase. Currently, MALDI ionization can support different types of mass analyzers, but the most common combination for proteomics studies is the MALDI/time-of-flight (TOF) setup [49]. In recent mass analyzers, ions generated in the source are accelerated to a fixed amount of kinetic energy and travel down a flight tube. The small ions have a higher velocity and are recorded by a detector before the larger ones. The m/z value displayed in a TOF spectrum is proportional to the time, for a given analyte, required to reach the detector. Unlike MALDI, the ESI source produces ions from the solution. Briefly, the ESI process consists of the formation of an electrically charged spray, driven by high voltage (2–6 kV), which triggers desolvation of peptide/protein-solvent droplets. This process is aided by high temperature and, in some cases, by sheath gas flow at the mass spectrometer inlet. There are different theoretical models to describe ESI ion formation, however the important features are: formation of multiply charged species, sensitivity to analyte concentration and flow rate.

Liquid chromatography (LC) instruments are usually coupled “on-line” with the ESI source to achieve continuous or high throughput analysis. For instance, reverse phase high-pressure liquid chromatography (RP-HPLC) has been widely adopted in proteomics to resolve very complex peptide mixtures prior to MS analysis (LC-MS), due to its high resolution, efficiency, reproducibility, and mobile phase compatibility with ESI. A further development of this technology is nano-ESI [50,51]. In this case, the flow rates are lowered to a nanoliter-per-minute regime to improve the sensitivity of the method. Nano-ESI is compatible with capillary RP-HPLC columns [52], allowing users to perform analyses with high sensitivity [53].

Two levels of information are provided by LC-MS analysis of peptides and proteins. First, molecular weight and the elemental composition of the analyte can be extracted when the analyzer achieves sufficient mass resolution. In the second, information about the primary sequence can be obtained if the peptide of interest (precursor ion) is subjected to tandem mass spectrometry (MS/MS) analysis. MS/MS is therefore a key technique for protein or peptide sequencing and PTM analysis. Collision-induced dissociation (CID) [54] has been the most widely used MS/MS technique in proteomics research. In this method, gas-phase peptide/protein cations are internally heated by multiple collisions with rare gas atoms. This leads to breakage of the C-N bond in the peptide backbone, resulting mainly in b- and y- fragment ions. However, CID fragmentation results in limited sequence information for large peptides (>15 amino acids) and intact proteins.

This limitation has been addressed by the development of novel methods for ion-electron reactions to carry out peptide fragmentation: electron capture dissociation (ECD) and electron transfer dissociation (ETD) enable sequencing of larger peptides, providing an option to investigate combinatorial features of hPTMs [55–58]. Both ECD and ETD are based on the transfer of electrons to the multi-protonated longer peptides (>2 kDa). In ECD, the electrons are generated from a heated filament composed of a rhenium-based alloy, whereas in ETD they are transferred by gas-phase radical ions. Despite the similarity between the two techniques, ECD can be used only in combination with fourier transform ion cyclotron resonance (FT-ICR) instruments, whereas ETD can be implemented in low-cost, high-capacity ion traps or new generation Orbitrap mass spectrometers and it has thus a wider applicability.

### 2.1. From “Bottom Up” to “Top Down”, via “Middle Down” MS Approaches in hPTM Research

The “Bottom Up” approach is highly popular in proteomics studies for investigations of protein PTMs [31]. It is a “peptide-centric” strategy, based on the enzymatic digestion of proteins into peptides prior to MS analysis. The “Bottom Up” approach has been demonstrated successful in identifying known and novel modifications on histones, combining its sensitivity in detecting peptide m/z in full MS with its efficient MS/MS fragmentation via CID [32]. The most common protease used in “Bottom Up” proteomics studies is trypsin, which cleaves at the C-terminal end of Arginine and Lysine residues [59]. However, trypsin digestion is not ideal for the analysis of histones that are highly rich in these basic residues (especially at the N-terminal regions, where the modifications accumulate), because the peptides produced are too short to be efficiently retained and separated in RP-HPLC and thereafter be detected by the mass spectrometer [60]. The endopeptidase Arg-C is a good alternative because of its specificity for the C-terminal region of Arginines, producing longer and easy-to-ionize peptides, also suitable for LC-MS [60,61]. In addition, peptides produced in this digestion retain a positive charge at C-terminal Arginine residues, leading to a well-defined y-ion series [62–64]. Alternatively, histones can be chemically derivatized using either propionic anhydride [(C_3_H_5_O)_2_O] or deuterated acetic anhydride (D6-acetic anhydride [(CD_3_CO)_2_O]), prior to trypsin digestion. These compounds trigger Lysine alkylation that prevents tryptic cleavages, resulting in Arg-C-like digestions. The advantage of this approach is that it leads to the described benefits of an Arg-C-like digestion while using trypsin as the protease, well-suited to in gel digestion [65]. The in gel approach, commonly performed by SDS-PAGE, facilitates separation at the level of individual histone molecules [66].

**Figure 1:**
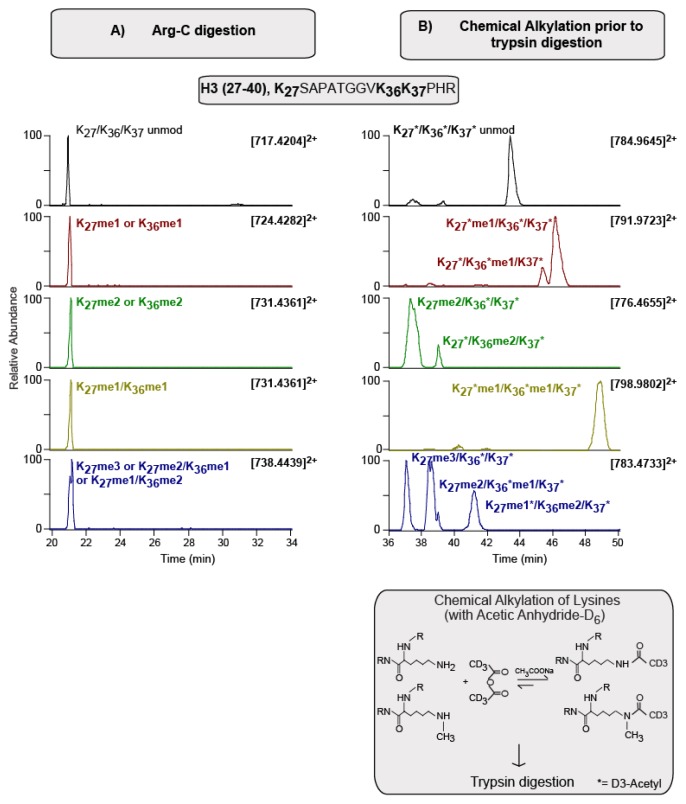
Elution profile of H3 (27–40) modified peptide. (**A**) Extracted ion chromatograms (XIC) of various 2+ charge modified forms relative to H3(27–40) peptide are reported, upon Arg-C digestion**,** for the time range corresponding to 20–34 min. Peptide ions at the specific *m/z* values: 717.4204, 724.4282, 731.4361 and 738.4439, correspond to unmodified, mono- (me1), di- (me2) and tri-(me3) methylated H3(27–40) peptides, respectively. In this case it is problematic to distinguish between methylations at K27 and K36; (**B**) Extracted ion chromatograms (XIC) of various 2+ charge modified forms relative to the H3(27–40) peptide are reported, upon deuterated acetic anhydride alkylation, prior to trypsin digestion, for the time range corresponding to 36–50 min (bottom panel describes the reaction in detail; the asterisk indicates the addition of a D_3_-acetyl group to unmodified and mono-methylated Lysine). Peptide ions at the specific *m*/*z* values 784.9645, 791.9723, 776.4655 and 783.4733 correspond to unmodified, mono- (me1), di- (me2) and tri-(me3) methylated H3(27–40) peptides, respectively. Peptide ion at 798.9802 *m*/*z* is assigned to mono-methylations at K27 and K36. Based on the number of D_3_-acetyl groups and methylations, distinct modification degrees at specific Lysines residues can be assigned unambiguously. With this strategy, distinct isobaric peptides (*i.e.*, K27me2 and K36me2) are resolved during chromatography by their discrete elution times.

Moreover, this derivatization labels unmodified and mono-methylated Lysines with a deuterated acetyl moiety (showing a delta mass of 45.0294 Da) but does not react with di-methyl, tri-methyl and acetyl Lysines, enabling the distinction between isobaric modification-bearing peptides. For example, peptide H3 (27–40) contains three Lysines, which can be differentially modified. In a case where two of these Lysines are mono-methylated, it is challenging to distinguish this species from an isobaric peptide containing a single di-methylation modification (Figure 1A). The derivatization approach however removes this isobaric feature (Figure 1B), since the addition of the deuterated acetyl moiety to unmodified and mono-methylated Lysines leads to a mass difference between the two-peptide isoforms. Furthermore, the peptides modified by distinct combinations of native and chemical modifications, display slightly different elution times, which contributes to the unambiguous assignment of modifications to specific residues.

A limitation of the “Bottom Up” approach emerges when analyzing histone variants or combinations of histone modifications. In fact, the short tryptic and Arg-C-like peptides do not permit detection of simultaneously occurring, long-distance PTMs. Offline chromatography, to separate histone variants or differently modified versions of the same histone molecule prior to “Bottom Up” analysis, is one solution to this problem. For instance, the three mammalian variants of histone H3 (H3.1, H3.2 and H3.3) share the majority of peptides produced upon enzymatic digestion; however the intact proteins can be separated prior to digestion and LC-MS using tap-tag purifications and/or RP-HPLC [67,68]. Alternatively, intact proteins or larger histone domains can be directly analyzed by mass spectrometry with the so-called “Top Down” and “Middle Down” strategies [69,70]. Histones are basic proteins and, in the acidic conditions used in MS, they are typically highly charged and thus capable of producing multiply charged fragment ions in MS/MS. Consequently, non-ergodic fragmentation methods [71] such as ETD and ECD on high-resolution instruments (Orbitrap, FT-ICR) are feasible for “Top Down” analysis [57,58]. “Top Down” enables the user to distinguish between co-occurring histone variants and differently modified isoforms, with information about the relative abundances and modification stoichiometries, thus providing a so-called “bird’s eye view” on the complete panel of histone isoforms present in a specific functional state [72]. The approach however lacks the sensitivity of “Bottom Up” and, furthermore, the analysis of the spectra obtained is less straightforward. These two restraints have limited the uptake of this approach so far, even though recent advances in online separation of intact proteins by ultra high-pressure (UPLC) liquid chromatography have made the approach more feasible. Further improvements in implementations are therefore still required to make “Top Down” analysis of intact histones, with variants and modified forms, a more routine approach [73,74].

The “Middle Down” approach is an optimal compromise between “Top Down” and “Bottom Up” strategies, when the mass spectrometer is hyphenated to online liquid chromatography. In “Middle Down” approach, large histone peptides (>2 kDa) are analyzed upon the enzymatic digestion of histones with endoproteinases that have specificities to less frequently-occurring amino acids within histone sequences, such as Glu-C or Asp-N. In fact, since mammalian H3 contains the first Glutamic acid at position 50, Glu-C at pH 8 produces an N-terminal peptide (1–50) of 6 kDa that contains the majority of PTMs decorating this histone, as well as being suitable for MS analysis and sequencing by either ETD or ECD MS/MS fragmentation. Similarly, Asp-N is useful for “Middle Down” analysis of histone H4, because it cleaves at the N-terminal side of Aspartic acid at position 24. Again, the resulting peptide (1–24) includes all modifications annotated at the H4 tail [33]. The “Middle Down” approach therefore allows a more precise detection of PTM combinations on particular histone regions, especially when combined with pre-fractionation of the enzymatic digestion products. For instance, a combination of weak-cation exchange with hydrophilic interaction liquid chromatography (WCX-HILIC) prior to high-resolution MS, efficiently resolve co-occurring and/or (near-) isobaric modified histone species [75], separating longer peptides first by their charge state and then by hydrophilicity. Based on this, Young *et al.* proposed a high-throughput approach using a gradient of decreasing organic solvent and decreasing pH on a commercial WCX-HILIC resin to separate and analyze by a “Middle Down” approach differentially modified histone domains [76] (See also Section 3).

**Figure 2:**
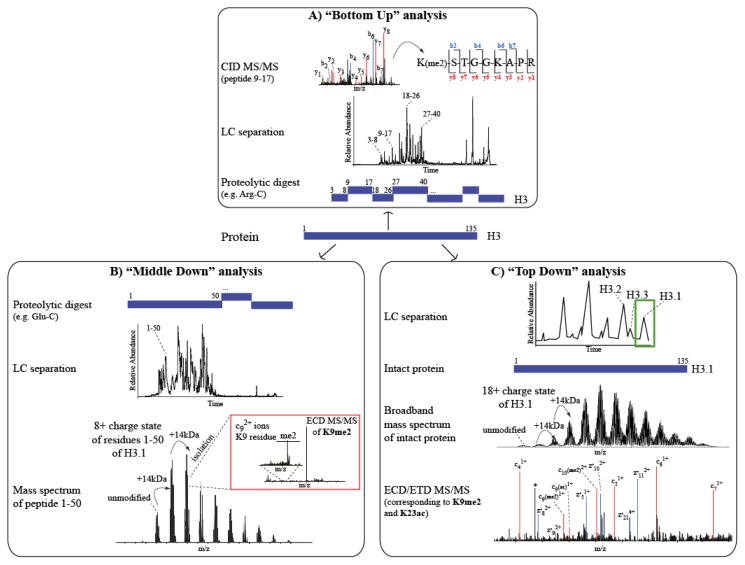
Comparison of “peptide-centric” *versus* “protein-centric” MS strategies for hPTMs analysis. (**A**) In a “Bottom Up” approach the H3 is first digested with Arg-C and the resulting peptides are subjected to LC–MS/MS analysis using CID fragmentation. The series of b- and y-ions generated permits the assignment of di-methylation on the K9 residue within peptide 9–17 of histone H3; (**B**) In a “Middle Down” approach the H3 is digested with Glu-C and the resulting peptides are subjected to LC separation. In the example, the full MS spectrum corresponding to peptide 1–50 is reported. The peak corresponding to 8+ charge state is then isolated and subjected to ECD fragmentation. Zoomed region (red) shows fragment ion c_9_^2+^, corresponding to K9me2; (**C**) RP-HPLC- purified intact histone H3.1 variant (green box) is directly MS analyzed in “Top Down” approach. In the example, the modified form of H3.1 is reported (middle panel) and the zoomed region of the ECD spectrum, corresponding to 18+ charge state of H3.1, is shown (bottom panel). K9me2 and K23 acetylation are identified on the same molecule through the characteristic c- and z-ion series produced via non-ergodic fragmentation.

An inconvenience of the “Top Down” and “Middle Down” approaches is the need for specialized software to summarize the complex combinatorial networks existing among hPTMs. The main problems concern the complexity of the MS/MS spectra generated, either from intact histones or from large peptides, and the increased incidence of internal peptide sequence fragments that further complicate the sequence annotation and consequently the PTM site-specific attribution in the MS/MS spectra [77–79] (Figure 2). Improvements in computational approaches should enable more detailed comprehension and visualization of the inter-reliant relationships between unique modified forms.

### 2.2. Bioinformatics Tools for hPTM Analysis

A number of bioinformatics tools have emerged to interpret the large amount of data generated by modern mass spectrometers. Of particular relevance to the analysis of the modifications that occur on histones are tools that enable identification of several different PTMs, often co-existing on the same peptide. Identification of PTM-bearing peptides in sequence databases, however, is more challenging than that of unmodified forms because the database search engine needs to take into account the diversity of modified forms that might exist. There are at present a number of computational methods available for the automated annotation of PTMs in peptides (Table 1). These methods analyze the MS and MS/MS data, taking into account the delta-mass values, and also neutral losses and other diagnostic ions for the PTM of interest [80].

The computational methods used to identify PTMs fall into two categories [32]. In the first, the user selects a set of PTMs of interest prior to employ the bioinformatics tool for peptide and protein identification. This option is applied during the sequence database search, when PTMs are assigned to the relevant amino acids of a candidate peptide sequence. To limit the complexity required to search a very large set of possible modified forms, a restriction is usually imposed on the number of modifications that may be included in this search.

In the second approach, which is unbiased, PTMs are identified through a “blind” database search. In the initial step, a basic database search is performed, excluding the specification of PTMs of interest, but often specifying recurring/standard modifications such as oxidized Methionine, for example. The specification of this relatively common modification avoids false-positive PTM assignments later on. Once a set of peptides is identified in an MS/MS-based proteomics experiment the idea is that, since the PTM leads to a mass increment or deficit of the modified peptide relative to the form without the modification present, all unassigned MS/MS spectra can be searched to find those which might match a post-translationally modified form. The software therefore inspects unassigned spectra, using information based on a list of known modifications such as delta-mass values and lists of predicted and observed peptide masses.

Computational methods that search for post-translational modifications are however associated with higher rates of false-positive identifications. The combinatorial issues linked with assigning the masses of included modifications can dramatically increase the number of peptide and protein candidates in the output. In this regard though, technological improvements that enable higher mass accuracy when generating the MS and/or MS/MS spectra have helped to address this issue [94]. High-resolution mass analyzers can resolve and identify peptides bearing modifications with very similar delta-mass values as well as multiply charged ions in MS/MS spectra. Recent data analysis software therefore considers product ions with multiple charges either before or during database searching.

Nevertheless some issues are still beyond the reach of current algorithms. The first is that some modifications may arise from *in vitro* artefacts rather than *in vivo* enzymatic activity. A well known example is the di-glycine (GG) tag which occurs on Lysine, and is used to determine ubiquitination sites: the elemental composition of this tag is identical to that of iodoacetamide (IAA), commonly used for the alkylation of Cysteines in standard shotgun MS proteomics workflows [95]. Another issue is that most of the available methods are sub-optimal for the analysis of MS/MS spectra deriving from long peptide sequences and intact proteins, which may result from “Top Down” or “Middle Down” approaches. As described in the recent review by Sidoli *et al.*[32], the complexity of these spectra requires more specialised search algorithms, which can efficiently determine monoisoptic peaks, recognize ion charge states and deconvolute multiply-charged ion signals into singly-charged ion mass values. Currently, only a few software packages are available for this purpose [32].

### 2.3. Quantitative MS-Based Approaches in Epigenetic Research

Various strategies have been developed in MS-based proteomics for accurate protein quantitation, from single proteins up to global proteome profiling. They can be grouped into four categories: chemical labeling, metabolic labeling, quantitation by the use of standard peptides and label-free. While the first three all rely on the use of differently isotope-encoded tags, the fourth implies the direct comparison among unlabeled proteomes. We refer to specialized reviews for an extensive description of these strategies for global protein analysis [96,97], while focusing on their application to the measurement of histone modification, variants and turnover.

Chemical derivatization as a means to modify cleavable residues has been widely applied in epigenetic studies for their technical advantages, previously described [62,63]. In addition, the alkylation of Lysines with the deuterated acetic anhydride can also be used to quantitatively estimate the acetylation status of histones. For instance, distinct acetylated forms of H4 in *Drosophila melanogaster* and their developmental changes have been profiled using D6-acetic anhydride prior digestion and MS-analysis [60]. Reinberg and co-workers, using propionylation of histones, demonstrated that a significant portion of nuclesomes are asymetrically modified in embryonic stem cells, mouse embryonic fibroblasts (MEFs) and HeLa cells with respect to two prominent histone modifications: H3K27 di-/tri-methylation and H4K20 mono-methylation [98]. Similarly, this strategy was used to observe the effect of G9a/Glp1 methyltransferase knockdown on global histone methylation [40].

Other chemical derivatization strategies, such as TMT and iTRAQ, have only been employed on chromatin for protein-level profiling, with no focus on PTM level changes [99–101].

*In vivo* metabolic labeling with isotope-encoded amino acids has emerged as the most powerful approach to accurately quantify changes of histones and their PTMs. In stable isotope labeling by amino acids in cell culture (SILAC), a growth medium is prepared where natural (“light”) amino acids are replaced by “heavy” SILAC amino acids. Cells grown in this medium incorporate the heavy amino acids. When light and heavy cell populations are mixed, they remain distinguishable by MS, and protein abundances are determined from the relative MS signal intensities [102]. The possibility offered by this strategy to combine two cell populations from distinct media at a very early stage of the MS-proteomics workflow, significantly reduces the effects of experimental variation in sample preparation, thus leading to very accurate quantitation, which only takes into account changes caused by the different functional states. In the last years, SILAC has gained wide popularity in proteomics and, more recently, also in chromatin studies [36,103–105]. SILAC is preferentially used to profile protein levels; however it has also been successfully applied to identify and quantify hPTMs, and in particular to profile modification dynamics during the cell cycle: Bonenfant *et al* showed increasing phosphorylation on histone H3 and H4 and decreasing methylation of H3K27/K36 during mitosis [106]; Pesavento *et al.* proved that H4K20 methylation degree was tightly linked to cell cycle progression while Scharf *et al.* demonstrated that H4K20 mono-methylation promotes chromatin assembly, facilitating the subsequent deacetylation of H4 [107,108]. Using a SILAC MS-based experiment, Jung *et al.* showed that Polycomb repressive complex Suz-12 promotes the establishment of H3K27 di/tri-methylation in mouse embryonic stem cells, with a functional interplay between H3K27 tri-methylation and H3K27 acetylation, functioning as molecular switch in this system [109]. A drawback of the SILAC approach is that it is limited to comparison of no more than three functional states in a single experiment [110]. Recently, however, our group circumvented this limit adapting the SILAC approach to a “spike-in” strategy to determine breast cancer-specific histone PTM signatures. In this study, we focused on human breast cancer and comprehensively analyzed PTMs on histones H3 and H4 from a panel of heavy-labeled cancer cell lines (MCF7, MDA-MB231, MDA-MB453 and T-47D). Their modification patterns were compared to unlabeled normal epithelial breast cells (MCF10), used as a “spike-in” reference. The “spike-in” SILAC approach enabled quantitative tracking of the modification changes in cancer cells, as compared to their normal counterpart. With the accuracy of this strategy, it was possible to identify PTMs specifically associated to distinct type of breast cancer cell line with different properties (aggressiveness/prognosis). Among them some were already known as modifications linked to cancer, such as a decrease of H4K20 tri-methylation, whereas some emerged as novel markers of breast cancer, such as reduced levels of H3K9 tri-methylation [111].

A further limitation of SILAC is that it cannot be directly applied to clinical samples, as it relies on metabolic labeling of actively dividing cells. However, an interesting recent trend is the use of SILAC as an internal standard in the so-called “super-SILAC” approach [112], providing a solution to this restraint. In the super-SILAC approach, a combined heavy-labeled proteome mixture is derived from different cell lines cultured in heavy-isotope media. This “standard” mixture can then be spiked into clinical samples [113], generating a universal reference for quantitation, similar to that used in microarray analysis. It is possible to envisage the applicability of this strategy to quantify hPTM patterns from clinical samples by generating a super–SILAC mixture prior to biochemical methods to purify chromatin regions and/or bulk histones, thus generating a comprehensive set of heavy-labeled histone peptides, containing virtually all known hPTMs, as a universal reference.

The SILAC method has also been adapted for a range of other applications. In pulse experiments SILAC was used to measure the turnover of both hPTMs and histone variants: Zee *et al.* showed that H2A.Z has higher turnover rates than canonical H2A variants and, more generally, that acetylated histone peptides appear to turn-over much faster than methylated ones [114]. A variation of SILAC, known as heavy methyl SILAC (hmSILAC), is used for high confidence identification of methylation at Lysines and Arginines. In heavy methyl SILAC labeling, ^13^CD3-Methionine is added to Methionine-depleted media; upon uptake in the cell, the “heavy” Methionine is converted into *S*-adenosyl Methionine (SAM), the sole donor of methyl groups in enzymatic methylation reaction. As such, histone and all non-histonic proteins that contain methylations are enzymatically heavy-methyl labeled. Such isotopically methylated peptides are then identified with high confidence in MS, based on the presence of the specific ‘light and heavy peak pair’ as marker of methylation, and subsequently quantified.

Ong *et al.* first used this strategy to identify unambiguously methylated sites *in vivo* on both histones and non-histonic proteins [115]. Afterwards, hmSILAC was applied to study the dynamic turnover of H3K9 tri-methylation in pericentric chromatin [116]. More recently, the same approach was applied to profile more globally the turnover of histone Lysine methylation, revealing that mono-, di-, and tri-methylated residues generally have progressively slower rates of formation. Furthermore, methylations associated with active genes were found to have faster rates than methylations associated with silent genes [117].

A combination of both standard- and heavy methyl- SILAC in pulse-chase experiments, carried out on synchronized cells, enabled Sweet *et al.* to track the progression of H3K79 methylations throughout the cell cycle [118]. In addition, it was observed that H3K79 mono-methylations from newly-synthesized H3 molecules have the same turnover rates as those in pre-existing histones, with no differences among the three H3 variants [118].

Label-free or ion intensity-based quantitation strategies have been applied in a few studies to profile differently modified, but isobaric histone isoforms, which have a special feature to present identical molecular weight/mass (isobars) but different PTMs configurations, so they are undistinguishable in full MS and can be hardly separated by standard LC. Since in MS/MS such isobaric species are distinguishable based on the positional selectivity of ion fragmentation, a relative quantitation is possible in a label-free MS/MS-based manner, using the relative ratios of their fragment ions. “Top Down” intact histone analysis was successfully used to quantify different modified forms of H3.2 and H4, in a label-free approach [119,120] (See also Section 3).

Lastly, synthetic, isotopically labeled peptides can be used as internal standards for both relative and absolute quantitation of histones and their PTMs in “spike in” assays. Briefly, isotope-encoded peptides are synthesized with the same sequence of the modified histone peptide of interest, derived from the endoproteinase digestion used in the study. Relative quantitation is obtained when a known concentration of the standard peptide is “spiked into” each histone sample from the panel under investigation, and the intensity of the each native modified peptide is compared with that of the standard. With the same approach the absolute quantitation of modified peptides can be also achieved, when a calibration curve of the ion intensity *versus* the peptide standard, injected at distinct concentrations, is calculated. Typically, this approach is combined with single or multiple reaction monitoring MS (SRM/MRM), enabling very sensitive detection of even sub-stoichiometric modifications. This technique benefits from the triple quadropole (QQQ) instrumentation. Briefly, targeted peptides are selected in the first mass analyzer (Q1), fragmented by CID (in Q2) and one or several of the fragment ions uniquely derived from the targeted peptide are measured by the third analyzer (Q3). In this way, each peptide is characterized by a specific “transition” which links both the precursor and fragment ions, observed in both analyzers. The identity of each peptide can be inferred from the “transition” and the relative abundance can be estimated from the transition intensity relative to that of the standard [121]. Darwanto and coworkers successfully employed SRM upon spike in of isotopically encoded histone peptides in U937 lymphoma cells expressing a mutated form of the hDot1a methyltransferase. They profiled changes in a set of hPTMs and observed that in these conditions the decrease of H3K79 methylation parallels a corresponding increase in H2B K120 ubiquitination [122].

## 3. Mass Spectrometry Analysis of Histone Variants and Their Modifications

In addition to post-translational modifications, histone variants contribute to the epigenetic regulation of gene expression [123]. Histone variants typically accumulate at specific genomic regions and show unique modification patterns, affecting a variety of chromatin-related processes. Some interpretative models propose that they represent an “extra layer” of the histone code [124], providing additional mechanisms to modulate chromatin structure. However, at least for the majority of variants, the processes by which specific variants accumulate at certain regions and are transmitted throughout the cell cycle remain unclear. Except for H4, all core histones and linker histones H1 have a number of variant counterparts, often differing in a few amino acids, which hampers their analysis via conventional approaches, such as antibody-based assays.

Mammalian histone H3 has three major variants (H3.1, H3.2 and H3.3), in addition to a testis-specific variant (H3t) and a centromeric variant (CENP-A). The major variants are very similar in sequence composition. Histone H3.1 differs from H3.2 by a change in Cysteine 96 to Serine, while H3.3 differs from H3.1 by only 5 residues. However, they display differences in their expression, enrichment at specific chromatin domains, and in their post-translational modification signatures. Studies of the PTM patterns of H3 variants have been performed, profiting from all MS approaches described: “Bottom Up”, “Top Down” and “Middle Down”. “Bottom Up” analysis of mammalian, *Arabidopsis thaliana,* and *Drosophila melanogaster* H3 variants revealed that H3.3 is enriched in modifications associated with transcriptional activity [125–127]. “Top Down” analysis of H3 variants from rat brains showed comparable results using this complementary approach [128]. Affinity purification of epitope-tagged H3.1 and H3.3 revealed a distinct set of modifications occurring on these two H3 variants before and after their assembly on chromatin, suggesting that pre-assembly modifications determine their final fate, as well as their PTM patterns on chromatin [67]. A combinatorial view of modifications on H3.1 and H3.3 from asynchronous or colchicine-treated HeLa was achieved by “Top Down”: with this approach it was observed that only 5% of K4 was mono-methylated and about 50% of K9 was di-methylated in the H3.1 pool from asynchronous cells. In addition, more than 90% of the H3.1 pool was acetylated: K14 and K23 represent the major sites of acetylation. Upon colchicine treatment, however, the unmodified, mono- and di- phosphorylated S10 and S28 were detected in a 2:3:1 ratio, in addition to the K9 methylation and acetylations described. The absence of the K4 methylation in the colchicine-treated samples was probably due to the relatively small pool of molecules containing this modification [72]. “Middle Down” analysis of H3 variants in a panel of rat tissues showed distinct patterns of H3.2 and H3.3 levels and modification status between various tissues [129]. “Middle Down” was also successfully applied to the identification of more than 200 modifications in H3.2 and 70 modifications in H4 from human samples, including several not previously described [76,120].

Canonical human histone H2A is encoded by sixteen genes in a genomic cluster. Kelleher and co-workers identified and characterized twelve unique sequences by using intact mass and fragmentation spectra [70]. The modifications on the canonical H2A are incompletely characterized: only phosphorylation of S1 and acetylation on the N-terminal K5 are reproducibly reported [130], as well as mono-ubiquitination at K119, involved in gene silencing and mediated by Polycomb proteins [131]. The non-canonical H2A variants include H2A.X, H2A.Bbd, H2A.Z and macro-H2A. H2A.X phosphorylated at S139 is the so-called gamma-H2A.X, which localizes to sites of DNA double strand breaks (DSB) in response to DNA damage and thus represents a mark of the DNA damage response (DDR). Acetylation and ubiquitination of H2A.X were also shown to be involved in this process: acetylation of K5 is a prerequisite for the poly-ubiquitination and the subsequent release of H2A.X from the DNA damage sites [132]. H2A.Z is present at promoters where it is believed to maintain active chromatin in regions adjacent to silent ones. However, potential roles in gene silencing have also been proposed [133]. Acetylation of K4 and K7 of this variant were identified by a “Middle Down” approach in Jurkat cells [130]. Macro-H2A, the largest H2A variant, is generally enriched at transcriptionally silent regions. MS characterization of macro-H2A identified K115 ubiquitination and S137 phosphorylation. The former is implicated in X-inactivation whereas the latter is enriched in mitosis [134,135]. In addition, K17 mono-methylation, K122 di-methylation and Y128 phosphorylation are identified [134].

A combination of CID and ECD MS fragmentation at protein and peptide levels led to the characterization of several H2B variants and associated PTMs [130,136]: acetylation on K5, K12, K15 and K20, and ubiquitination on K120. These PTMs were confirmed by peptide mass fingerprinting (PMF) MS analysis on bovine H2B, which revealed also K43 mono-methylation and K85 acetylation [137]. “Bottom Up” approaches have also served to characterize modifications specific for the testis-specific variants of H2B (TH2B) [138]. In addition, “Top Down” analysis using ECD fragmentation of the two major H2B variants of *Tetrahymena thermophila* led to the characterization of their primary sequences and modification patterns [139]. Recently, mono-methylation and di-methylation at the N-terminal Proline of *Drosophila melanogaster* H2B have been identified using a combination of different strategies for sample preparation prior to MS analysis including D6-acetic anhydride derivatization followed by Trypsin digestion and Asp-N digestion. The abundance of this Proline methylation seems to depend on the developmental stage and is regulated by the enzyme dART8. The authors also observed predominant acetylation of H2B at K11 and K17 [140].

Histone H1 is commonly referred to as the linker histone. A single copy of this histone is proposed to bind near the entry/exit site of DNA on the nucleosome (the so called dyad), stabilizing the 30 nm fiber and thus regulating higher order chromatin structure and stability. Sequence divergence between histone H1 isoforms occurs mainly in the *N*- and *C*-terminal regions of the proteins, generating as many as eleven mammalian isoforms.

Mass spectrometry contributes to the identification of a number of PTMs specifically enriched on distinct linker histones, such as methylation, phosphorylation, acetylation, ubiquitination, formylation and ADP ribosylation [141–145]. RP-HPLC of the different H1 variants, followed by chemical derivatization of the protein with propionic anhydride and subsequent LC-MS/MS analysis revealed a K26 methylation and S27 phosphorylation on histone H1.4. Methylation on K26 appears to recruit heterochromatin protein 1 (HP1), whereas phosphorylation at S27 seems to inhibit HP1 binding, so that these two adjacent PTMs are believed to function as a molecular switch to modulate gene silencing [143,146]. Moreover, “Top Down” analysis of intact H1.2 and H1.4 purified at distinct cell cycle stages provided indications that S173 on H1.2 and S187 on H1.4 are phosphorylated only during interphase. Interphase phosphorylated H1.2 and H1.4 associate to active rDNA genes to facilitate their RNA Pol I-mediated transcription. Finally, phosphorylation of H1 reduces its association to chromatin and, consequently, the accessibility to factors that regulate transcription and replication [147].

## 4. Interaction Proteomics to Study Chromatin Architecture

**Figure 3:**
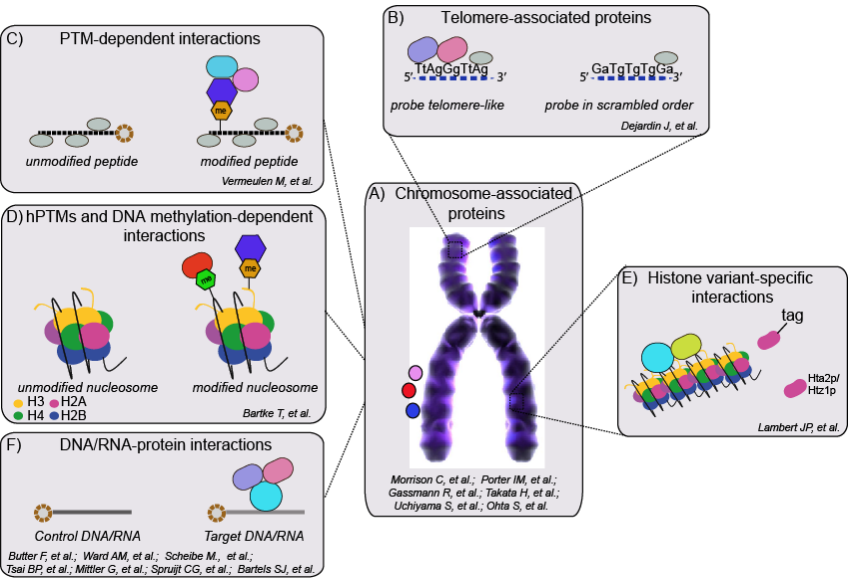
Different biochemical approaches for the proteomic characterization of chromatin architecture. (**A**) Some strategies address the chromosome as a whole, whereas others focus on the characterization of specific chromatin regions: (**B**) telomeres, regions enriched with modifications on (**C**) histones or (**D**) DNA and (**E**) specific histone variants. (**F**) Finally, some approaches aim at characterizing protein associating to chromatin via binding to distinct RNAs molecules and/or DNAs sequences.

A better knowledge of chromatin composition can contribute to a more comprehensive view of its higher-order structure and function. Until now, no purification method has emerged as a “gold standard” for chromatin purification and characterization, due to the difficulty in enriching chromatin samples from specific functional regions at a purity and quantity sufficient for subsequent analysis. In spite of these limitations and thanks to recent achievements in MS-based proteomics in terms of sensitivity and accuracy of quantitative information, a number of studies have demonstrated the high potential of this technology to characterize the chromatin proteome, with a focus on the histone code readers associated with specific functional regions (Figure 3).

An analysis of changes in protein levels in response to the overexpression of the oncoprotein MYC was the first attempt to characterize chromatin-binding proteins. This was achieved using differential detergent/salt extraction and chemical isotopic labeling by ICAT, in combination with multi-dimensional chromatography and mass spectrometry [148]. Subsequently, when *ad hoc* biochemical protocols were established for the purification of distinct chromosomes, MS proved to be successful in characterizing their protein composition: mitotic chromosomes were purified at different stages of the cell cycle (mitosis, metaphase and interphase) and the associating non-histone proteins were identified by MS [149–154]. More recently, a multiclassifier combinatorial proteomics (MCCP) approach was developed, where SILAC quantitative proteomics is integrated with a bioinformatics analysis pipeline. A statistical approach is applied to confirm which known and uncharacterized proteins are chromosomal, to obtain a more comprehensive and unambiguous collection of proteins associated with mitotic chromosomes [155].

One elegant methodology to study the proteomic composition of telomeric regions was developed by the Kingston group using the PICh (*Proteomics* of *Isolated* *Chromatin*) approach. In this method, enrichment of cross-linked telomeric chromatin was achieved using DNA probes complementary to the telomeres, rich in repetitive sequences. The co-enriched proteins were characterized by MS and new telomere-associated proteins were observed [156]. Yet, a drawback of PICh is the limited applicability to regions rich in repetitive DNA sequences.

All these methods provide a useful contribution to the knowledge of protein composition in large chromosomal regions or even intact chromosomes, but they are inadequate for gaining information on chromatin locus-specific composition.

Recently, a number of interactomics assays combining affinity-interaction mapping with SILAC-quantitative MS read-out have been developed for the comprehensive characterization of hPTM “readers”. Vermeulen *et al.* used pull-down assays with peptides that differ by a single post-translational modification to identify specific binders, either as individual interactors or as multiprotein complexes. With this approach, they discovered that TFIID binds H3K4 tri-methylation and recruits the entire transcription initiation complex, thereby providing a functional link between this modification and activation of transcription [157]. The approach was extended further to screen all major tri-methylation marks on histones and, in combination with ChIP-Seq and BAC-GFP pull-downs, to define the comprehensive Lysine trimethyl-interactome [158]. As an additional elaboration of the strategy, a SNAP (*SILAC Nucleosome Affinity Purification*) approach was established where recombinant nucleosomes bearing combinations of hPTMs and methylated DNA were used as baits to provide a “modification binding profile” for proteins regulated by the contribution of both DNA and histone methylations [159]. Similarly, a SILAC-based affinity purification assay was carried out with recombinant, uniformly modified chromatin templates [160]. In addition, the CLASPI (*Cross-Linking Assisted and SILAC-based Protein Identification*) approach has been described, which combines SILAC with chemical proteomics using photo-crosslinking-based histone peptide probes, to detect weak but specific interactions that may escape standard pull-down approaches [161]. Finally, peptide arrays and MS have been employed to systematically uncover methyl-Lysine and chromatin-binding module interactors, as well as to identify novel H3K23 mono-methylation mark that mediates the recruitment of HP1 eta to heterochromatin [162]. Tandem affinity purification (TAP) was also used to purify and identify chromatin-associated complex: such strategy has been successful employed recently to characterize PRC1 (Polycomb Repressive Complex 1) complexes [163].

These *in vitro* studies are very powerful tools for screening the soluble binders of hPTMs, but fall short in extracting information on the relative PTM stoichiometry, their combinations, and their synergies with histone variants and chromatin modifiers, under physiological conditions. Hence, the locus-specific determination of hPTM patterns and their interactions with protein complexes remains a very attractive, partially unachieved goal.

A SILAC-based quantitative proteomics approach was employed to generate a differential profile of proteins associated with both euchromatin and heterochromatin, exploiting the different accessibility of these regions to MNase, as a consequence of the differential nucleosome packaging. Upon limited MNase treatment, the two fractions of chromatin were separated by centrifugation, based on the differential density of the nucleosomal stretches; SILAC was used to discriminate the proteins associated with these two functional chromatin regions [164]. Another approach developed for detection and characterization of proteins associated with specific chromatin domains is mChIP [165], where chromatin is isolated, sheared by sonication and then MS analysed. mChIP was successfully applied to study the interactomes of H2A (Hta2p) and its variant Htz1p in *Saccharomyces cerevisiae*. However, this study did not provide quantitative information on binding proteins, and thus had limited ability to discriminate specific binders from nonspecific chromatin-associated proteins. Recently, an approach to characterize the proteins and hPTMs associated with a specific genomic locus was described, combining *Chromatin Affinity Purification and Mass Spectrometry* (ChAP-MS). A single genomic LexA DNA binding site was utilized to purify and characterize the GAL1 locus, under transcriptionally active and repressive conditions [166]. Building on these approaches, our group has recently developed a global, quantitative proteomic strategy, named ChroP (*Chromatin Proteomics*), to characterize functionally distinct chromatin regions [167]. Native and Cross-linked chromatin immunoprecipitation, combined with SILAC-based quantitative proteomics, permit global investigations of synergies between histone PTMs, variants, and chromatin modifiers associated with silent and active chromatin regions, marked by H3K9 tri-methylation and H3K4 tri-methylation. We identified previously characterized protein associations and also revealed numerous novel interactions suggesting original pathways on chromatin. Among them, the histone variant H2A.X and the chromatin remodeling complex WICH were both found enriched in heterochromatin, suggesting that their specific recruitment by H3K9me3 may represent an additional level of modulation of the DNA damage response (DDR) in this chromatin compartment. The implementation of ChroP is relatively straightforward, given the minor modifications to the standard ChIP protocol for ChIP-Seq; hence, it is amenable to numerous applications in various functional studies by epigenetics groups, to dissect chromatin composition and dynamics in a system-wide fashion.

DNA methylation also plays a pivotal role in mediating the epigenetic inheritance of specific expressions pattern on the genome, and the study of the interacting proteins is critical for a deep understanding of the molecular basis of its function. This has been achieved using immobilized oligonucleotides in combination with quantitative proteomics, in a setup very similar to protein-based pull down assays: DOC-1 (Deleted in Oral Cancer-1) and RBP-J (recombination signal binding protein for immunoglobulin kappa J region) were found to bind methylated CpG regions with this strategy [168–170]. In line with studies that aim to link the genome features with their associated protein interactome, two recent studies found a novel protein interactor of a single nucleotide polymorphism at the IGF2 locus, which may provide an explanation for the phenotypic effects of this polymorphism [171,172].

Finally, given the increasing importance of RNA in the epigenetics field [173,174], the combination of RNA-pull-downs with quantitative proteomics enable investigations at both the RNA-protein interactome and protein-protein interactions mediated by the presence of non-coding RNAs [175,176]. Typically, protein UV cross-linking followed by immunoprecipitation (CLIP) or RNA-binding protein immunoprecipitation (RIP) in combination with microarray hybridization or sequencing provide transcriptome-wide identification of interacting RNAs [177]. Alternatively, mass spectrometry can be employed to screen systematically for proteins specifically bound to a specific RNA recognition element (RRE) within a longer RNA fragments. This quantitative approach has been used to characterize proteins binding to the 3′-untranslated region (3′-UTR) of HDAC2 [175] and of the viral gene DENV-2 [178]. Recently, purification of cross-linked MS2-tagged ribonucleoproteins, using SILAC for unambiguous discrimination of genuine binders, has also been described [179]. The extension of such protein-centric approaches to other RNA-centric methods will allow the genome-wide detection of novel RNA-binding proteins (RBP) for distinct RNAs species.

## 5. Conclusions

Mass spectrometry-based proteomics has emerged as a powerful analytical method for the analysis of histone proteins, their post-translational modifications and variants, as well as their associated “writers” and “readers”. The method is therefore able to provide information that is in line with, and highly complementary to, other commonly used techniques, such as ChIP-Seq. In this review we have provided an overview of various MS-based proteomics approaches that enable novel insights into chromatin biology. In recent years, the advances in the dynamic range and sensitivity of MS instruments have allowed for improved detection of sub-stoichiometric histone PTMs. Furthermore, the novel chromatin interactomics’ studies described in this review have led to the identification of regions specific modifications, variants and non-histonic associated proteins.

Detecting the combinatorial aspect of the histone code, however, remains a daunting task. The “Bottom Up” approach is efficient for amino acid sequencing and improved throughput for complex samples, however, it offers only a partial view of the complex cross-talks occurring among different hPTMs. In this respect, advances in “Middle Down” and “Top Down” approaches suggest that only a combination of the three MS approaches will provide the comprehensive solution to crack the histone code. At present however two major limitations limit the applicability of protein–centric approaches: first, the lack of straightforward “Middle Down” or “Top Down” analytical workflows, from sample preparation and protein separation, to MS detection and data processing, compatible with large-scale analyses; secondly, the bioinformatics tools available do not effectively handle the higher complexity of tandem mass spectra.

The global and quantitative profiling of hPTMs and the cognate interactomes by MS may have a strong impact in cancer epigenetics research. In fact, while for a long time much effort has been invested in identifying genetic mutations in cancer, in recent years the scientific community has progressively recognized that, in some cancers, epigenetic components may predominate over the genetic ones, with genetic and epigenetic determinants of cancer initiation and progression intricately entwined. In this light, the deep understanding of the epigenetic aberration in cancer is essential to recognize better why cancers arise (because the factors that could cause genetic damage might not be the same as those that could cause epigenetic damage) and why some cancers may respond better to certain types of therapies, as some types of therapies may be more efficient on epigenetically-damaged cancers. In this light, strategies based on the combination of high-resolution MS and quantitative proteomics for the analysis of chromatin, represent reliable, comprehensive and sensitive tools for the detection of changes in PTM abundances during the transition from a healthy condition to the tumor, offering an essential contribution to the understanding of epigenetic phenomena in cancer biology.

## Figures and Tables

**Table 1:** Software and search algorithms used to study hPTMs.

Software	Freely available	Unbiased PTM search	Reference
FindMod	+	−	[81]
Mascot	−	−	[82]
MaxQuant	+	−	[83]
Modificomb	+	+	[84]
OMSSA	+	−	[85]
Phenyx	−	−	[86]
PILOT PTM	+	+	[87]
ProSightPTM 2.0	+	+	[88]
Protein Prospector	+	+	[89]
QuickMod	+	+	[90]
SEQUEST	−	−	[91]
SIMS	+	+	[92]
VEMS 3.0	+	−	[80]
X!Tandem	+	−	[93]
